# Advancing public health workforce’s professional development: implications for Ukraine

**DOI:** 10.1093/eurpub/ckaf143

**Published:** 2025-08-19

**Authors:** Olga Gershuni, Martina Parić, Olga Aleksandrova, Rok Hrzic, Timo Clemens, Genc Burazeri, Nataliia Piven, Matt Commers, Katarzyna Czabanowska

**Affiliations:** Department of International Health, Care and Public Health Research Institute (CAPHRI), Faculty of Health, Medicine and Life Sciences (FHML), Maastricht University, Maastricht, The Netherlands; WHO Collaborating Centre on Public Health Leadership and Workforce Development, Department of International Health, Care and Public Health Research Institute (CAPHRI), Maastricht University, Maastricht, The Netherlands; Department of International Health, Care and Public Health Research Institute (CAPHRI), Faculty of Health, Medicine and Life Sciences (FHML), Maastricht University, Maastricht, The Netherlands; WHO Collaborating Centre on Public Health Leadership and Workforce Development, Department of International Health, Care and Public Health Research Institute (CAPHRI), Maastricht University, Maastricht, The Netherlands; World Health Organization, Country Office in Ukraine, Kyiv, Ukraine; Department of International Health, Care and Public Health Research Institute (CAPHRI), Faculty of Health, Medicine and Life Sciences (FHML), Maastricht University, Maastricht, The Netherlands; WHO Collaborating Centre on Public Health Leadership and Workforce Development, Department of International Health, Care and Public Health Research Institute (CAPHRI), Maastricht University, Maastricht, The Netherlands; Department of International Health, Care and Public Health Research Institute (CAPHRI), Faculty of Health, Medicine and Life Sciences (FHML), Maastricht University, Maastricht, The Netherlands; WHO Collaborating Centre on Public Health Leadership and Workforce Development, Department of International Health, Care and Public Health Research Institute (CAPHRI), Maastricht University, Maastricht, The Netherlands; Department of International Health, Care and Public Health Research Institute (CAPHRI), Faculty of Health, Medicine and Life Sciences (FHML), Maastricht University, Maastricht, The Netherlands; Department of Public Health, Faculty of Medicine, University of Medicine, Tirana, Albania; World Health Organization, Country Office in Ukraine, Kyiv, Ukraine; Department of International Health, Care and Public Health Research Institute (CAPHRI), Faculty of Health, Medicine and Life Sciences (FHML), Maastricht University, Maastricht, The Netherlands; WHO Collaborating Centre on Public Health Leadership and Workforce Development, Department of International Health, Care and Public Health Research Institute (CAPHRI), Maastricht University, Maastricht, The Netherlands; Department of International Health, Care and Public Health Research Institute (CAPHRI), Faculty of Health, Medicine and Life Sciences (FHML), Maastricht University, Maastricht, The Netherlands; WHO Collaborating Centre on Public Health Leadership and Workforce Development, Department of International Health, Care and Public Health Research Institute (CAPHRI), Maastricht University, Maastricht, The Netherlands

## Abstract

Despite many challenges, Ukraine has prioritized the need to develop and professionalize the public health workforce (PHWf) to respond to its transitioning public health (PH) system. This study explores how various PH stakeholders can develop and advance the PHWf professionalization programme in Ukraine using the WHO-ASPHER Roadmap to Professionalize the PHWf in the European region as the theoretical foundation. A mixed-methods qualitative approach was used to (i) document analysis of Ukrainian policy and regulatory documents related to PHWf and (ii) qualitative survey research involving relevant PH stakeholders. Directed and conventional content analysis was applied to analyse data. The document analysis reviews key areas as LoPHS, PH higher education, and continuous professional development. The analysis helped explain the current state of the PHWf and to understand enabling factors for its professionalization. Results from the qualitative survey research suggest several actions to enhance PHS in Ukraine based on three priority areas: (i) PH laws and regulation; (ii) PH education and training; and (iii) financing for the development and professionalization of PHWf. The PHWf professionalization programme for Ukraine resonates with the WHO-ASPHER Roadmap to Professionalizing the PHWf in the European region. It attempts to close the gap between PH legislation, the current state of the PHWf, and international PH practice ambitions. The study suggests the need for a well-coordinated development process across PHS and PHWf domains.

## Introduction

Stimulated by the adoption of the Law ‘On Public Health System’ (LoPHS) (Zakon Pro Systemy Hromadskhgo Zdorovia) in 2022, Ukraine initiated a process of professionalization of its public health workforce (PHWf) [1]. The Ukrainian government strives to ensure a robust public health system (PHS) and deliver all essential PH functions (EPHFs). In Ukraine, medical services (except of vaccinations) are not considered part of the PHS, and there is no division between core and wider PHWf. The country currently struggles with a variety of large-scale challenges, ranging from healthcare and PHS reform and the invasion by Russia, to the increasing burden of non-communicable diseases, mental health morbidity, and environmental impact. The ongoing war has a profoundly detrimental effect on the infrastructure, healthcare system, and service delivery in general [2, 3]. Assuring comprehensive and sustainable capacities to deliver essential PH functions and services is essential. Yet PHWf development, recognition, planning, and monitoring remain underdeveloped and vulnerable [4].

### The WHO-ASPHER Roadmap to Professionalizing the PHWf in the European region

The professional world is evolving with growing workloads, new technologies, the way professionals work and are perceived [5]; What you need to become a professional depends on the field and it means certain education, following specific standards and codes of conduct [6]. There are attempts to describe PHWf professionalization worldwide, however, such processes are often inconsistent, and clarity is missing [7]. The WHO-ASPHER Roadmap to Professionalizing the PHWf in the European region (hereinafter WHO-ASPHER Roadmap) provides pragmatic recommendations for professionalization and addresses capacity-building requirements to address PH challenges. The Roadmap is helpful at governmental, organizational, and professional levels as it highlights the need for a coordinated effort to support PHWf [8]. A unified approach also calls for comprehensive measures that help professionalize PHWf, tailored to the specific situation and resources available [9].

### Public health infrastructure, reform and challenges in Ukraine

In 2014–2015, Ukraine initiated the development of a new PHS in accordance with the Sustainable Development Strategy ‘Ukraine-2020’ and the EU-Ukraine Association Agreement. Ukraine sought to establish a PHS responsive to health emergencies and promoting population health, guided by a 2015 EU evaluation and legislative commitments [10]. The Cabinet of Ministers adopted the PHS Development Concept in 2016, followed by the approval of the Action Plan for 2017–2020 in 2017. Moreover, in 2021, Ukraine pursued a public health assessment to obtain an objective evaluation of its adherence to International Health Regulations [11]. A significant step was taken in 2022 by adopting LoPHS, which provides a foundation for meeting international best practices and workforce standards modernization [1, 12]. It outlines EPHFs and identifies institutions at the national (PH Centre of the Ministry of Health of Ukraine—UPHC) and regional levels (‘oblast’ Centers for Disease Control and Prevention—oCDCs). Additionally, the oCDCs will help coordinate other stakeholders’ delivery of PH functions. This new role is challenging for oCDC leadership, given a dearth of PH professionals in the regions with the skills needed to promote PH, coordinate preparedness, and manage emergency responses [13].

Professionalization of PHWf presents a challenge to Ukraine. Due to the war, Ukraine faces substantial relocation and outflow of healthcare and PH professionals. This has created a severe shortage of expertise, posing a threat to PH services delivery and, thus, to communities [14]. High turnover means knowledge loss and challenges in maintaining standards and therefore threatens professionalization. The shortage of qualified personnel will remain a significant problem after the war and require prudent decision-making.

As Ukraine strives to achieve its ambitions in PHWf professionalization despite historical disadvantages and the pressures of war, the country will need to prioritize carefully. Therefore, there is a pressing need for clearer understanding of the status of PHWf efforts in Ukraine.

This study explores how PH stakeholders in Ukraine can effectively develop and promote the PHWf Professionalization Programme within the country’s PHS. The WHO-ASPHER Roadmap will function as a theoretical foundation to support identification of priority areas and strategies for PHWf professionalization in Ukraine [8]. Study findings constitute the basis for an actionable plan and recommendations.

## Methods

The researchers triangulated [15] data from two qualitative methods: (1) document analysis to review the current PHWf-related regulatory and policy situation in Ukraine and (2) qualitative survey research with stakeholders assessing needs, expectations, barriers, and facilitators to PHWf professionalization in Ukraine. Two forms of qualitative content analysis [16] were applied: directed content analysis using predetermined categories from the WHO-ASPHER Roadmap [8] and conventional content analysis identifying emergent categories from the data [16].

### Qualitative mixed methods

#### Document analysis

Document analysis is often used in health policy studies to improve qualitative analysis and understanding of situations [17, 18]. The researchers thoroughly examined documents and analysed reports, policy documents, and regulations related to the development of PHWf to understand the current status of PHS in Ukraine according to official resources. The following documents were analysed: LoPHS, standards of higher education in the speciality PH for bachelor’s, master’s level, and scientific level; Ministry of Health of Ukraine (MoH) Order on ‘Internships under Martial Law’; Ministry of Health of Ukraine Order for the ‘Approval of Internship Regulations’; Cabinet of Ministers of Ukraine Resolution for the ‘Approval of the Regulation on the System of CPD of Medical and Pharmaceutical Workers’. The data were structured according to three predetermined categories: LoPHS, standard in PH education, and continuous professional development (CPD).

#### Qualitative survey research

Qualitative survey research supports exploring the diversity of opinions, experiences, and expectations [19]. This approach is beneficial in assessing needs, experiences, and barriers faced by different stakeholders involved in professionalization processes [19]. Qualitative survey research comprised (1) closed pre-interview and (2) open questionnaire. A total of 19 participants completed a written closed pre-interview form outlining essential professionalization leavers and measures regarding development and implementation in Ukraine based on the WHO-ASPHER Roadmap (Supplementary Material S1). The research team also designed an open questionnaire for interviews (Supplementary Material S2). The researchers conducted 11 virtual, semi-structured interviews. The identical open questions were provided on paper to respondents who opted for written responses. A total of eight participants provided written answers. Interviews were carried out in English and Ukrainian. Interviews conducted in Ukrainian were transcribed in Ukrainian with simultaneous translation into English. Interviews were recorded with permission using videoconferencing software. Transcripts of interviews and written responses were anonymized and categorized as: (i) policymakers, (ii) implementers, and (iii) academia. Only one researcher (not involved in data analysis) possessed an identification number for each respondent.

Stakeholder sampling was purposeful based on predetermined criteria [20]. Inclusion criteria included: all stakeholders must be related to the core PHWf (consistent with the WHO-ASPHER Roadmap [8]) within PHS as framed by LoPHS [12] and related to policy formulation. Additionally, participants were involved in implementing EPHFs or providing PH training in academic institutions. Respondents were identified by the MoH and fell into three categories: policymakers at the MoH, the State Institution ‘Public Health Centre of the MoH of Ukraine’ (n = 4), implementers at the Centres for Disease Control and Prevention of the MoH of Ukraine (n = 7), and academicians (n = 8). Data collection occurred in June–July 2023 and was organized by the WHO Country Office in Ukraine. The research team analysed data from 19 participants.

### Qualitative content analysis

First, the researchers conducted directed content analysis that creates codes based on existing theory [16]. The researchers coded the data from closed pre-interviews according to the following predetermined categories: (1) key professionalization leavers: competencies, training and education, formal organization, professional credentialing, and code of ethics & professional conduct; and (2) professionalization measures: alignment of PH services with EPHOs, PH laws and regulations, financing for PHWf, PHWf taxonomy, job descriptions, PHWf data, enumeration, planning, and forecasting, PHWf recruitment and retention strategies, CPD, core competencies for PHWf. ATLAS-ti23 software was used.

Secondly, conventional content analysis for open questionnaire was applied. This approach is less structured than directed content analysis allowing coding categories to emerge from the text. Analysis utilized non-predetermined categories to summarize the views and perspectives of the stakeholders [16]. The blended data, combined with the WHO-ASPHER Roadmap professionalization and measures [8], enabled the formulation of recommendations for sustainable PHWf development and professionalization in Ukraine.

Participants signed an informed consent form, and the research was submitted to the Research Ethics Committee at Maastricht University to audit the study.

## Results

A qualitative mixed-methods of document analysis and qualitative survey research comprised the results. The analysis of official reports, policy documents, legislation, and regulations on PHWf development in Ukraine was conducted. Insights from the stakeholder perspective were included to provide an understanding of the current status of PHWf.

### Current state of the PHWf in Ukraine (document analysis)

#### The (2022) LoPHS

This legislation’s primary objectives are to promote health, prevent diseases, enhance quality-of-life, and prolong life expectancy. LoPHS does not define PHWf (or its division to core and wider workforce), but it outlines PHS and defines key institutions within it [12, 21]. The Cabinet of Ministers of Ukraine ensures development and implementation of PHS's state policy. The Cabinet coordinates organizational, staffing, financial, and scientific support for PHS and ensures the conditions required for its development. The MoH of Ukraine is the central executive body that ensures the formation, organization, and implementation of state policy in the healthcare field. It also approves PHWf development program in the PHS. The State Institution PH Center of the MoH of Ukraine provides training, retraining, and advanced training of human resources within the PHS. Several sections describe the functions and powers of PH Centre of the MoH of Ukraine, with aspects interlinked with performance of PH operational functions and needed competencies. A dedicated section on staffing and scientific support of the PHS identifies specialties involved in PH provision (e.g. ‘Public Health’, ‘Epidemiology’, and ‘Hygiene’). However, it provides only general requirements for developing human resources and education standards. Unlike many other European countries, PHS in Ukraine does not include medical services provision.

#### Higher education standards in public health: The Ministry of Education and Science of Ukraine

The Ministry of Education and Science of Ukraine approved standards for the master’s degree program in PH (MPH) in December 2018 [22]. Thereafter, several universities obtained licenses from the Ministry of Education and Science to offer MPH programs in 2019. The first state exam for these MPH programs took place in 2021. The PH Bachelor’s and PhD education standards were approved in 2020 and 2022, respectively. The Ministry of Education and Science of Ukraine [23] provides a detailed overview of educational standards and learning objectives within PH field, focusing on training specialists to perform complex PH tasks. PhD graduates are additionally expected to conduct research and perform various pedagogical activities [24].

#### Regulation of CPD

The Ukrainian Law on professional development of the employees outlines how employees’ qualifications are checked to match the skills and knowledge required for their roles, assessing professional level through credentialing [25]. The aim is to help employers use labor efficiently and ensure adequately skilled employees. Regulation of the CPD system for healthcare workers mandates that they engage in ongoing professional development [26, 27]. From 2024, all employees listed in the Directory of Qualification Characteristics of Occupations of Employees (Issue 78) ‘Healthcare’ are part of the CPD system, including professionals with non-medical education [28, 29]. However, the process for certifying specialists with non-medical education, such as PH specialists, has not yet been established, leaving the CPD pathways for PHWf unclear.

### Ukraine’s current state of public health and PHWf (qualitative survey research)

Participants identified the areas for improving PHWf and strengthening PHS in Ukraine. First, participants ranked priority areas based on predetermined categories (key professionalization leavers and professionalization measures) derived from the WHO-ASPHER Roadmap, using a scale of 1–5 (Table 1). Next, the data from the open questionnaire identified emergent categories. Based on the qualitative content analysis, the combined results converged according to the top three identified priority areas: (1) PH laws and regulation, (2) PH education and training, and (3) financing for development and professionalization of PHWf. Participants acknowledged barriers, gaps, and needs related to PHS and PHWf development in Ukraine and identified the main stakeholders that should be involved with professionalization (Supplementary Material S3).

**Table 1.** Average of priority areas according to the WHO-ASPHER Roadmap ranked on a 1–5 scale based on the responses from nineteen participants

Having PH laws and regulations	5,125
PH education and training	4,875
Securing financing of the development and professionalization of the PHWf	4,875
Continuing professional development	4,75
PH competencies	4,625
Core competencies for specific areas of PH (e.g. field epidemiology, laboratory health protection, NCDs and CDs, environmental hazards, occupational health, etc)	4,625
Developing PH job taxonomy and later job descriptions	4,25
Enumerating the PHWf (how many, who making an inventory, creating a database, etc)	4,125
Aligning the PH services and functions with WHO EPHOs	4,125
Developing recruitment and retention strategies	4
Code of ethics and professional conduct	3,875
Credentialing and licensing	3,875
Accreditation	3,75
The formal organization of a profession (chamber)	3,125

#### Priority area 1: public health laws and regulation

Although LoPHS legally frames PHS’ structure and functions, it is difficult to interpret corresponding implementation arrangements. It was indicated that better communication and collaboration mechanisms between stakeholders, and access to evidence-based materials and best practices, would improve the situation. Participants acknowledged that the abovementioned actions demand good governance and systems thinking, which, when applied, should strengthen PH in Ukraine.

#### Priority area 2: public health education and training

Most participants agreed that ensuring funding for PH research and education is essential. Investments in PH education and training, with an eye toward employability and needed competencies, would benefit PHWf professionalization and build capacity. Respondents asserted that some PH educational institutions are too focused on epidemiology and laboratory skills and should focus on developing multifaceted, interdisciplinary PH professionals instead. A clearer connection among PH education, employment market, and competencies necessary for PH within the Ukrainian context would improve the employability of graduates and make the field more attractive for (young) professionals. CPD should be available for PHWf focusing on topics such as epidemiology, crisis response, leadership, and managerial competencies. Additionally, participants highlighted limited digital skills, inequitable workforce distribution in urban and rural areas, lack of leadership and recognition by the public, and the developing labor-market.

#### Priority area 3: financing of the development and professionalization of PHWf

The development of PHS and PHWf in Ukraine faces several challenges, including an aging workforce, inadequate funding, and uncompetitive remuneration. Creating a detailed national PH development strategy for PH would support a more unified approach to PHWf professionalization. Lack of data for decision-making, including PHWf development and enumeration, hinders planning for and delivery of essential PH services. CPD should be available for PHWf focusing on topics such as epidemiology, crisis response, leadership, and managerial competencies. Collaboration with international stakeholders (including WHO, ASPHER, CDC, and ECDC) can support designing effective and efficient development strategies and thus attract financing for further enhancement of PH education and professionalization.

## Discussion

For countries developing and professionalizing PHWf, clarity is crucial. The importance of a transparent and specific definition of PHWf cannot be overstated [30]. It prevents harmful confusion, and helps ensure that PH professionals are respected and valued. The PHWf in Ukraine does not have a clear legal definition that would help identify the responsibilities; PHWf is therefore unprepared to anticipate and shape implementation of LoPHS. This suggests the questionable official status of PHWf as a profession in Ukraine and the lack of legislative literacy relating to PH functions and services. This study identified an urgent need to ‘unpack’ LoPHS and to encourage dialogue among policymakers, implementers, and academics on how PHS is shaped and should be explained to PHWf. Additionally, incorporating legal topics within bachelor and master curricula could ensure that students become literate about legal frameworks, health policy, health governance, regulatory compliance, and related ethical considerations. Incorporating legal topics within undergraduate and graduate programs could potentially support the early adoption of those principles into the practice and support alignment of legal aspects with key focus areas in PH.

Before adopting LoPHS, PH functions on a regional level were spread across institutions. Now, within LoPHS, oCDCs play a key role in implementing EPHFs as the first-ever institution at regional level responsible for the entire spectrum of PH functions, including disease prevention, health protection, and health promotion. A major challenge for an effective PHS at regional level is the shortage of qualified personnel needed to implement the oCDCs’ functions in immunization, antibiotic resistance, infection control, health promotion, non-communicable diseases, and health communication [13].

The Joint External Evaluation of IHR Core Capacities of Ukraine (2021) highlighted several areas important for human resource development. The assessment of training needs to ensure PHWf preparation for PH emergencies directly relates to PHWf development. Also, the formal agreements between PH and other sectors (e.g. agriculture, defence, and foreign affairs) to facilitate cooperation and information exchange should be developed [13].

A significant challenge in PHWf development is ensuring that professionals engage with appropriate credentialing, certification, and licensing systems, as these directly influence guidance into training programs and CPD [31]. While professional regulation ensures that PHWf meets specific standards, often through mandatory licensing, credentialing verifies a professional’s ability to deliver quality services. Effectively addressing these issues will result in a skilled, adaptable workforce and help maintain professional motivation [32]. Although credentialing and professional regulation must align with real-life practice, credentialing and licensing currently do not apply to PHWf in Ukraine as a non-medical profession. Improving Ukraine’s PHWf is therefore a key priority, even as the country implements practical solutions [33, 34].

Unlike other European countries [35], PHS in Ukraine does not include medical services provision, which can affect the understanding of PHWf provided in this study. The CPD activities described by the Law and the Regulations on CPD [25, 26, 28] are primarily focused on developing medical practices to meet patient needs and improve efficiency. This focus poses additional challenges for the professionalization of the PHWf.

The PH field in Ukraine needs investments in infrastructure and technology to make CPD programs more accessible and effective. Although the country has many higher educational institutions, the diversity of program formats and the increasing number of PH graduates do not necessarily stimulate optimal PHWf development. Career paths for PH graduates are unclear, and the profession suffers from a deficit in status and prestige. To address this, training programs need standardization, including CPD programs, credentialing, specialization tracks, mentorship programs, and field placements [9, 30, 32]. There is a pressing need for significant strengthening of the competence profiles of PH professionals through, for instance, the application of existing competency frameworks within the Ukrainian context [4, 36, 37].

PHWf professionalization is a complex process without a uniform strategy [6, 7]. It is often influenced by reforms, legislation, events like the COVID-19 pandemic, and geopolitical instability. Despite those challenges, there are examples of how countries can support their PHWf. In Ukraine, the UPHC and National Health Service of Ukraine [38, 39] are registered as CPD providers and provide free e-platforms for PH and healthcare workers training.

Information on the current state of PHWf in Ukraine, its priority areas, the impact of facilitators, and barriers on professionalization can be used to support appropriate strategies toward strengthening and developing PHWf [40]. There is a pressing need to engage policymakers and stakeholders to prioritize policy upgrades and investments in strengthening and developing the PHWf in Ukraine.

### Recommendations

Information gathered from stakeholder interviews allows for identifying prioritized needs for PHWf professionalization in Ukraine. Table 2 is aligned with the professionalization leavers and measures of the WHO-ASPHER Roadmap and provides recommendations based on the qualitative survey research results, consolidated with the top three priority areas: (1) comprehensive and uniform implementation of PH laws and regulations; (2) update, revise, and reform PH education and training by focusing on enhancing PH in several meaningful ways; and (3) securing governance and financing of the development and professionalization of the PHWf.

**Table 2.** Recommendations for the development of the PHWf Professionalization Programme in Ukraine

1. Comprehensive and uniform implementation of PH laws and regulations
The law on PHS already provides the framework, but it is essential to ensure that this framework aligns with the comprehensive policy needed for implementing the EPHFs in the country Develop national definitions for PHWf and define the list of PH services within the country’s jurisdiction Promote the dissemination of a list of PH services and create a collaborative environment for their understanding and implementation Establish a national minimum dataset for the PHWf that can be used to standardize practice and assist in monitoring and quality assurance Establish the enumeration and forecasting mechanisms and develop retention strategies for PHWf Establish a unified policy for PHWf development and professionalization Strengthen the implementation and alignment with WHO's 12 EPHFs within country-specific requirements and the current state of PHS Reveal and address critical gaps in the PHWf capacity by conducting regular needs assessments Promote data-driven decision-making in PH, emphasizing data collection, analysis, quality assurance, and tools like Health System Performance Assessment (HSPA) and Health Technology Assessment (HTA). Ensure PHWf is trained in evidence-informed policymaking Develop quality indicators for PH services and provide quality assurance and improvement training. Prioritize quality assurance training for PHWf to uphold the highest standards
2. Update, revise, and reform PH education and training by focusing on enhancing PH in several meaningful ways:
Develop a national PH competencies framework aligned with European and international standards Increase collaboration with academic partners and international organizations Commit to translating professional best practices effectively for local PHWf Emphasize the employability of PH trainees Invest in research to strengthen PH education that supports and informs teaching methods and educational processes Establish research centers dedicated to bolstering the professionalization of PHWf Develop CPD programs: from short courses to comprehensive programs
3. Securing governance and financing of the development and professionalization of the PHWf:
Secure adequate funding to support the growth and professionalization of the PHWf Establish clear national-level strategic goals for PH to guide collective efforts Measure progress by developing national indicators to gauge the performance of the PH system Establish a coordination mechanism to ensure effective and smooth collaboration among PH professionals across various sectors Establish strong governance structures and processes in PH, ensuring transparency, accountability, participation, and integrity (including the following areas: reporting duties; public information; conflict of interest policies/organizational separation; codes of conduct; consultation processes with the public and other sectors; advisory committees; clear legal mandates; audits, etc) Clarify task allocation between different administrative levels within the PH field Enhance collaboration and communication skills for effective stakeholder engagement Empower PH professionals to advise on legislation and policies, raise awareness of legislation, and operate effectively within the legal framework Develop leadership capacities at all administrative levels to ensure adequate capabilities within PH Integrate PH leadership, law, and governance modules into basic training and continuing education programs

## Conclusion

The strategy to develop and implement the Ukrainian PHWf Professionalization Program is derived from synthesizing the evidence from document analysis and qualitative survey analysis. It provides practical answers to the question: “How can PH policy stakeholders in Ukraine develop and implement the PHWf professionalization program?” Ukrainian stakeholders can navigate complex organizational change by following several accelerators, ensuring PHWf professionalization is initiated, prioritized, and sustained. An integrated policy framework can enhance coordination, streamline efforts, and maximize resources to respond effectively to a broad spectrum of PH challenges. This will require appropriate collaboration and communication among government agencies responsible for PH delivery (such as Ministries of Justice, Education, Health, etc) and actively involve related stakeholders (such as emergency and professional associations). The requirements for PHWf should include forming clear timelines, responsibilities, and PH practice performance indicators. The process should be divided into phases with regular assessments and adjustments to ensure effectiveness and sustainability.

### Limitations

Though this study can inform policy and practice related to PHWf professionalization in Ukraine, there are limitations regarding generalizability. PHS in Ukraine does not include medical services and does not clearly define its PHWf. While Ukraine’s recent reforms and distinct PH context offer valuable insights, therefore, the specific policy implementations mentioned may not apply to other countries without careful consideration and adaptation. The researchers cannot guarantee that the stakeholders interviewed were evenly distributed and possessed equal expertise in PH law, epidemiology, health prevention, promotion, and protection. This potential bias may influence the ranking of the priority areas and the distribution of the codes related to barriers, needs, and gaps. The questionnaire included areas such as accreditation, credentialing, and licensure as professionalization leavers but provided no explanation, leaving them somewhat open to interpretation. It can be assumed that the respondents meant institutional accreditation when they were asked to rank the accreditation component. Although the participants suggested that the priority areas (Table 1) should be developed simultaneously, the research team focused on three priority areas according to the stakeholder’s preferences. Given the evolving field of PH in Ukraine and the availability of recourses related to the PHWf, the authors acknowledge a possibility of missing information that emerged after data collection in 2023.

## Supplementary Material

ckaf143_Supplementary_Data

## Data Availability

The authors confirm that all data supporting the findings and recommendations of this study are provided in the article and the supplementary files. Key points The study used the WHO-ASPHER Roadmap to Professionalizing the public health workforce (PHWf) in the European region as a theoretical background. The development and implementation of the Public Health Competency Framework and Continuous Professional Development Framework for Ukraine is an appropriate step toward professionalizing the country’s PHWf. Ukrainian public health policymakers, implementers, and academic stakeholders must actively collaborate and engage to prioritize investments in strengthening the PHWf. The development and implementation of a national approach to enumerating and forecasting the PHWf will support public health services at the administrative, organizational, and community levels. Developing executive public health leadership programs and ‘unpacking’ the Law ‘On Public Health System’ will ultimately benefit the aligned delivery of Essential Public Health functions and strengthen the PHWf capacity.
