# Supplementary material for: Advancing public health workforce’s professional development: implications for Ukraine
Source: Eur J Public Health. 2025 Aug 19;35(6):1150–5. doi: 10.1093/eurpub/ckaf143 (PMC12707465; doi:10.1093/eurpub/ckaf143)
Supplement: ckaf143_Supplementary_Data [file ckaf143_supplementary_data.docx]

**Supplementary Materials**

**Supplementary Material 1. Pre-interview guide. Professionalization of the Public Health Workforce in Ukraine.**

The following guide will help you prepare for the interview. Expect to spend approximately 20 minutes preparing. Please, read and reflect on the questions in part I. After, please fill in the survey in part II.”

| 1. **Guiding questions** |
| --- |

| 1. Defining the scope of the public health workforce |
| --- |
| - How will you define the scope of the public health workforce in your country? |

| - Which professionals are part of the public health workforce? - What is their composition? (quantitative^[[1]](#footnote-1)^ and qualitative^[[2]](#footnote-2)^) - Are they likely to change in the future? How such a change can affect the public health workforce? |
| --- |

| 1. **Inventory Survey** |
| --- |

Below, you will find a checklist of necessary leavers and measures that need to be developed to support the professionalization of the Public Health Workforce in Ukraine.

Tables 2a, 2b aim to help you identify the specific areas for development or improvement and should help in identifying the priority areas.

| **Table 2a. Assessment of professionalization leavers** | | | | | | |
| --- | --- | --- | --- | --- | --- | --- |
|  | **DEVELOPED** | | | **NOT DEVELOPED** | | |
| **Professionalisation levers** | Public Health degrees | | | Public Health degrees | | |
|  | Bachelor | Master | PhD & CPD | Bachelor | Master | PhD & CPD |
| Public health education and training (academic) |  |  |  |  |  |  |
| Competencies |  |  |  |  |  |  |
| Accreditation |  |  |  |  |  |  |
|  | Within medical specialization | Independent  For PH | Other | Within Medical specialization | Independent  For PH | Other |
| Credentialing and licensing |  |  |  |  |  |  |
| Formal Organisation |  |  |  |  |  |  |
| Code of Ethics and Professional Conduct |  |  |  |  |  |  |

| **Table 2b. assessment of professionalization measures status** | | | | | | |
| --- | --- | --- | --- | --- | --- | --- |
|  |  | | |  | | |
|  | **DEVELOPED** | | | **NOT DEVELOPED** | | |
| **Professionalization measures** | Developed and implemented | Developed but does not function | Developed but needs a small adjustment | Does not exist and currently not possible to develop | Awareness of the need and some preliminary talks | The feeling of urgency and will to develop |
| Alignment of PH services with EPHOs |  |  |  |  |  |  |
| Public health laws and regulations |  |  |  |  |  |  |
| Financing for public health workforce |  |  |  |  |  |  |
| Public health workforce taxonomy |  |  |  |  |  |  |
| Job descriptions |  |  |  |  |  |  |
| PHW data, enumeration, planning and forecasting |  |  |  |  |  |  |
| PHW recruitment and retention strategies |  |  |  |  |  |  |
| Continuing Professional Development (CPD) |  |  |  |  |  |  |
| Core Competencies for PH Workforce |  |  |  |  |  |  |

1. **Prioritization of actions**

***What will you start with? What is your priority? and Why?***

**Supplementary Material 2. The professionalization of the PHW in Ukraine Interview Guide.**

1. How would you define the PHW in Ukraine?

a. Which professionals are part of the public health workforce?

b. What is their composition? (where do they work and what do they do)

c. What sort of education do they have?

2. Who is responsible for the provision and evaluation of PH services in Ukraine?

3. What are in your opinion some gaps concerning the provision of these services?

4. What do you think should be the aims of the professionalization of the PHW in Ukraine? What could the professionalization of the PHW achieve? Can you please motivate your answer?

5. We have shared with you a Pre-interview guide. Part II of the guide (Inventory) includes professionalization leavers and measures, which are necessary to be developed to assure the development of a strong and well-recognized public health workforce. These include:

1. PH education and training
2. PH Competencies
3. Accreditation
4. Credentialing and licensing
5. Formal organization of a profession (chamber)
6. Code of Ethics and Professional Conduct
7. Continuing Professional Development
8. Developing PH job taxonomy and later job descriptions
9. Enumerating the PHW ( how many, who, making an inventory, creating a database), etc
10. Developing recruitment and retention strategies
11. Core competencies for specific areas of PH (eg. Field epidemiology, laboratory, health protection NCDs and CDs, environmental hazards occupational health….)
12. Aligning the PH services and functions with WHO EPHOs
13. Having PH laws and regulation
14. Securing financing of the development and professionalization of the PHW
15. What are the priority areas according to you? What would you recommend to do first and why?

6. Which stakeholders can support you in addressing these priority areas? Who has interest and power, and who can potentially oppose?

7. If you would like to implement these priorities, what will be the main barriers and facilitators that you will have to address, and why?

8. What are some topics that are important for the professionalization of the PHW in your specific context that we have not discussed, in your opinion?

**Supplementary Material 3. An overview of the main codes identified during the analysis: barriers, needs, and gaps (visualized in codes).**

| **Barriers** | **Needs** |
| --- | --- |
| old school' approaches  ageing workforce  current image of PH  current ongoing reform  digital skills & implementing digital innovation  hierarchy in decision making  inadequate funding  inequitable distribution of PHWf (rural/urban)  lack of appropriate legal framework  lack of quality control of CPD  lack of strategic understanding & political determination  legislative conflicts  losing graduates to other sectors  no clear functional division of structures  no regulations to enforce PH legislation  no PHWf statistics available  uncompetitive remuneration  unwillingness to change & working independently  varying stages of reform  changing PHWf composition | wide range of specialised knowledge  CPD  access to evidence-based materials  better salaries  competency assessment for high ranking officials  data for planning services sytematically  developing and implementing PH legislation  education & training for English language  education & training for high ranking officials  eHealth systems  facilitate communications between stakeholders  funding for PH research  implementation protocols on quality standards  multidisciplinary teams & intersectoral community  matching education with PHWf needs  media support & public awareness  national indicators & definitions for PH  PH leaders at regional levels  shift towards prevention  state policy for PHWf development  systematic approach in different administrative territories  unified approach in different oblasts |
| **Stakeholders** |  |
| (international) associations & professional associations  academic institutes and universities  Cabinet of Ministers of Ukraine (CMU)  centres for disease control and prevention  community development specialists  EU and Member States  health centres & healthcare facilities  international institutions  medical universities  MoH  oblast  professionals  Public Health Center  social protection departments  State Service of Ukraine on Food Safety and Consumer Protection  WHO  health departments  medical staff  PH professionals  regional information & analytical centres |  |
|  | **Gaps** |
|  | knowledge translation  intersectoral collaboration within the systems and public/private  crisis communication  PHW training in medical and non-medical universities  no control at macro level  no environmental service  occupational health and safety  operationalization of professional PH associations  understanding quality assurance & management  srategic national plan for PH  effective communication  use of media and public awareness  leadership in PH  “One Health” approach application  multi-stakeholders engagement in developing regulations  leadership training  managerial competency  systematic international collaboration |
| **Barriers** | **Needs** |
| old school' approaches  ageing workforce  current image of PH  current ongoing reform  digital skills & implementing digital innovation  hierarchy in decision making  inadequate funding  inequitable distribution PHW (rural/urban)  lack of appropriate legal framework  lack of quality control of CPD  lack of strategic understanding & political determination  legislative conflicts  losing graduates to other sectors  no clear functional division of structures  no regulations to enforce PH laws  no PHW statistics available  uncompetitive remuneration  unwillingness to change & working independently  varying stages of reform  changing PHW composition | wide range of specialised knowledge  CPD  access to evidence-based materials  better salaries  competency assessment for high ranking officials  data for planning services sytematically  developing and implementing PH legislation  education & training for English language  education & training for high-ranking officials  eHealth systems  facilitate communications between stakeholders  funding for PH research  implementation protocols on quality standards  multidisciplinary teams & intersectoral community  matching education with PHW needs  media support & public awareness  national indicators & definitions for PH  PH leaders at regional levels  shift towards prevention  state policy for PHW development  systematic approach in different administrative territories  unified approach in different oblasts |
| **Stakeholders** |  |
| (international) associations & professional associations  academic institutes and universities  Cabinet of Ministers of Ukraine (CMU)  centres for disease control and prevention  community development specialists  EU and Member States  health centres & healthcare facilities  international institutions  medical universities  MoH  oblast  PH centrers' laboratories & laboratory professionals  Public Health Center  social protection departments  State Service of Ukraine on Food Safety and Consumer Protection  WHO  health departments  medical staff  PH professionals  regional information & analytical centres |  |
|  | **Gaps** |
|  | knowledge translation  collaboration public/private  crisis communication  lack of PHW training in medical universities  no control at macro level  no environmental service  occupational health and safety  operationalisation of professional PH associations  understanding quality assurance & management  strategic national goal & definitions for PH  effective communication  use of media and public awareness  leadership from MoH  integrated “One Health” approach  involve relevant stakeholders in developing regulations  leadership education  managerial competency  systematic international collaboration |

1. Capacity, demand, ratio with population, in-/ outflow, activity rates [↑](#footnote-ref-1)
2. Training background, age, competencies, self-identification, morale, motivation(s), recognition (professional/social), EPHO alignment, inclusive, intersectoral and multidisciplinary, skills-mix … [↑](#footnote-ref-2)
